# Integration of *Ixodes ricinus* genome sequencing with transcriptome and proteome annotation of the naïve midgut

**DOI:** 10.1186/s12864-015-1981-7

**Published:** 2015-10-28

**Authors:** Wibke J. Cramaro, Dominique Revets, Oliver E. Hunewald, Regina Sinner, Anna L. Reye, Claude P. Muller

**Affiliations:** Department of Infection and Immunity, Luxembourg Institute of Health (former Centre de Recherche Public de la Santé)/Laboratoire National de Santé, Luxembourg, Luxembourg

**Keywords:** *Ixodes ricinus*, Genome, Transcriptome, Proteome, *de novo* assembly, Annotation, Midgut, Lyme Borreliosis

## Abstract

**Background:**

In Europe, *Ixodes ricinus* ticks are the most important vectors of diseases threatening humans, livestock, wildlife and companion animals. Nevertheless, genomic sequence information is missing and functional annotation of transcripts and proteins is limited. This lack of information is restricting studies of the vector and its interactions with pathogens and hosts. Here we present and integrate the first analysis of the *I. ricinus* genome with the transcriptome and proteome of the unfed *I. ricinus* midgut.

**Methods:**

Whole genome sequencing was performed on *I. ricinus* ticks and the sequences were *de novo* assembled. In parallel, I. ricinus ticks were dissected and the midgut transcriptome sequenced. Both datasets were integrated by transcript discovery analysis to identify putative genes and genome contigs were screened for homology. An alignment-based and a motif-search-based approach were combined for the annotation of the midgut transcriptome. Additionally, midgut proteins were identified and annotated by mass spectrometry with public databases and the in-house built transcriptome database as references and results were cross-validated.

**Results:**

The *de novo* assembly of 1 billion DNA sequences to a reference genome of 393 Mb length provides an unprecedented insight into the *I. ricinus* genome. A homology search revealed sequences in the assembled genome contigs homologous to 89 % of the *I. scapularis* genome scaffolds indicating coverage of most genome regions. We identified moreover 6,415 putative genes. More than 10,000 transcripts from naïve midgut were annotated with respect of predicted function and/or cellular localization. By combining an alignment-based with a motif-search-based annotation approach, we doubled the number of annotations throughout all functional categories. In addition, 574 gel spots were significantly identified by mass spectrometry (*p* < 0.05) and 285 distinct proteins expressed in the naïve midgut were annotated functionally and/or for cellular localization. Our systems approach reveals a midgut metabolism of the unfed tick that is prepared to sense and process an anticipated blood meal.

**Conclusions:**

This multiple-omics study vastly extends the publicly available DNA and RNA databases for *I. ricinus*, paving the way for further in-depth analysis of the most important European disease vector and its interactions with pathogens and hosts.

**Electronic supplementary material:**

The online version of this article (doi:10.1186/s12864-015-1981-7) contains supplementary material, which is available to authorized users.

## Background

*Ixodes ricinus* is the most common tick species and the most important vector of human and animal pathogens in Western Europe [1, 2]. Ixodes ticks are obligate hematophagous ectoparasites of vertebrates with a single blood meal at each development stage of their life cycle from larvae to nymphs and adults. During feeding, they transmit a range of pathogens, mostly bacteria (e.g. *Borrelia burgdorferi* sensu lato (s.l.), *Anaplasma* spp., *Rickettsia* spp.), but also viruses (e.g. tick-borne encephalitis virus) and protozoa (e.g. *Babesia* spp.). Globally, they are only second to mosquitos as disease vectors with serious consequence for human health. They also effect productivity and/or welfare of livestock, wildlife and companion animals. With 85,000 cases diagnosed annually and a high number of unreported cases, Lyme borreliosis (LB) is the most important vector-borne disease in Europe. LB is a multisystemic disease with a pathognomonic erythema migrans, that develops in 60 - 80 % of infections with *Borrelia*. The incubation period can be as long as 36 days [3]. Unspecific symptoms like headache, fever or fatigue are frequent. If the early stage of the disease is not treated appropriately, Lyme arthritis, Lyme neuroborreliosis or Acrodermatitis chronica atrophicans may develop [4]. In Luxembourg, 11 % of *I. ricinus* ticks are infected with human pathogenic members of the *Borrelia burgdorferi s.l*. complex [5] which is slightly below the overall infection rate of 14 % in Europe [6]. *Borrelia* bacteria live within the midgut of the tick and migrate, after primary blood uptake, to the salivary glands from where they are transmitted to the host [7]. Interactions between *I. ricinus* and *Borrelia* in the tick’s midgut are essential for survival of the pathogen in the tick and its transmission to the host. Thus, midgut proteins are the important players in vector-pathogen interactions and present potential targets for blocking transmission, e.g. by vaccines. Vaccination of the host could induce antibodies that could potentially interfere with vital functions of the midgut already during the early feeding stage. The only commercially available tick vaccine, which is directed against the cattle tick *Rhipicephalus microplus*, is indeed based on a midgut protein [8]. Since tick midgut proteins are normally hidden from the host immune system, they have no immunomodulatory effect on the host [9]. Many midgut proteins are involved in the digestion of blood proteins. In arthropods, proteases and peptidases involved in blood digestion were found to be encoded in paralogs, which are expressed under different environmental conditions and developmental stages [10, 11]. In ticks, a dynamic multi-enzyme network responsible for hematophagy has been identified in the partially and fully engorged midguts [12, 13]. The presented sequences will now allow for further investigation of the presence of digestive enzymes already in the unfed midgut and paralogous genes encoding digestive enzymes in *I. ricinus*. Due to climatic change [14, 15], afforestation and increasing deer populations [16], the number of ticks and the risk of tick bites is on the rise in Europe. Since ticks and tick-borne diseases become more and more important for human and animal health, understanding of tick-host and tick-pathogen interactions is essential. The development of mitigation strategies is complicated by weak and incomplete databases for ticks in general and *I. ricinus* in particular. Therefore, genomic, transcriptomic and proteomic data with robust annotation of their function and localization are urgently needed as publicly available reference databases. To fill this gap, we combined genome and transcriptome sequencing with proteome analysis by peptide mass fingerprinting - providing the first insight into the genome of *I. ricinus* as well as annotation of putative function and cellular localization of transcripts and proteins in the naïve *I. ricinus* midgut.

## Results and discussion

### Genome sequencing and *de novo* assembly

As a first step towards the whole genome of the tick *I. ricinus*, 998,100,906 paired-end reads were obtained from an Illumina HiSeq™ 2500 sequencer. The 99,810,090,600 bp obtained correspond to a theoretical 47 fold coverage, assuming a genome size similar to the ~2.1 Gb of *Ixodes scapularis*, the American relative of *I. ricinus*. 613,376,348 reads (61 %) were assembled into 235,953 contigs with a minimum size of 1 Kb. 384,724,558 reads remained as singletons. The N50 size was 1,643 bp and the N75 size was 1,257 bp. The longest contig had a size of 32,538 bp. Mismatches within reads and contigs during back-mapping of the paired-end reads to the assembled contigs identified potential local sequence errors (e.g. short insertions or deletions) as well as structural errors (e.g. scaffolding errors). Accordingly, sequences were error-corrected, whenever necessary. The assembled contig sequences as well as the raw sequencing reads are publicly available at the National Center for Biotechnology Information (NCBI) (JXMZ01000000 and SRP051465, respectively). Altogether, the contigs span a length of 392,924,918 bp, which corresponded to 19 % of the putative genome size of ~ 2.1 Gb. The algorithm is not able to assemble short sequence repeats to large contigs as it is not possible with short read lengths to distinguish between high sequence coverage and large domains of short sequence repeats. So the assembly of repeats with a repeat unit larger than the length of the reads cannot be solved. Therefore, the assumption was made that mostly unique sequences were covered in their full length by the assembly and that repetitive regions may be covered only once per repeat. This hypothesis is supported by the results from a redundancy analysis, revealing that there are no highly similar contig sequences in our genome assembly. According to Ullmann et al. [17], only 30 % of the *I. scapularis* genome consists of unique DNA sequences. If the *I. ricinus* genome has a similar organization, our contigs would cover 63 % of unique sequences. This estimate is in line with our finding of 67 % of midgut mRNA reads mapping to the assembled genome contigs. Since the midgut is a major organ of the *I. ricinus* tick, we may assume that a high percentage of both, house-keeping transcripts and organ-specific transcripts were covered by our transcriptomic data. Compared to the 26–27 % of *I. ricinus* RNA reads from adult females and larvae that could be mapped against the *I. scapularis* genome reference by the Genomics Resources Development Consortium [18], the more than doubled percentage of mapped reads further underlines the need for and the utility of an *I. ricinus* reference genome also for transcriptome and proteome analyses. Direct mapping of our genome contigs against the *I. scapularis* genome assembly as a reference revealed that 54 % of our contigs aligned with at least 80 % identity to *I. scapularis* scaffolds, whereas 46 % showed less similarity. We found homologous sequences to 89 % of the *I. scapularis* scaffolds in our *I. ricinus* contigs, indicating coverage of most genome regions by our assembly, even if coverage is partial (Additional file 1). Since there is no annotation of paralogs for the *I. scapularis* genome available, no conclusion on the coverage of paralogous sequences can be drawn from this homology analysis. However, the mapping of 67 % of all mRNA reads to 79 % of our genome contigs (Additional file 2) is another strong indication of a broad coverage of most genome regions. Thereby we demonstrate, that the assembled genome contigs do not only represent a minor part of the non-repetitive genome consisting of a small set of highly expressed genes, but rather a broad spectrum of unique genome regions with various expression levels (indicated by diverse total read counts per genome contig). Furthermore, complementary genome sequences for all *I. ricinus* genes in NCBI Genbank were identified in our assembly (Additional file 3). Vice versa, 19,744 genome contigs (8 %) represented sequences that did not match our homology criteria against *I. scapularis* (Additional file 1). A homology comparison of the latter sequences to all Acari genomes available at NCBI Genbank excluding *I. scapularis* (namely *Dermatophagoides farinae*, *Metaseiulus occidentalis*, *Rhipicephalus microplus*, *Tetranychus urticae* and *Varroa destructor*) revealed 13 contigs with homology to one of the further related species. Thus, with few exceptions, contigs without homology to *I. scapularis* did not show homology to any other sequenced Acari species, but were rather unique to *I. ricinus* (Additional file 4).

### Identification of putative genes in the assembled genome contigs

In order to identify putative open reading frames and genes in the *I. ricinus* genome, 10,039,099 midgut mRNA reads of average 118 bp lengths were mapped with a large gap read mapper to the above assembled genome contigs. The different techniques by which the genomic and mRNA reads were obtained (Illumina and IonTorrent) do not interfere with the integration of both individual data sets by mapping and alignment, because these methods are not affected by technique-specific error types or rates. For performing analyzes that could be critical to this issue, e.g. *de novo* assembly, the datasets were strictly separated. The large gap read mapper algorithm is able to map RNA-Seq reads that span introns without prior transcript annotation by allowing large gaps in the mapping. It is therefore also suitable for transcript analysis in non-annotated reference genomes. For transcript discovery, the position of each gap in the read mapping was analyzed with respect to its location at a valid splice site. These mapping events were analyzed for proximity to identify events that are close enough to belong to the same gene (= coverage region). Events that supported the same splice sites were merged. Events with unaligned ends shorter than 17 bp were retained if they were within 9 bp of an observed splice site and were fixed to these sites. This fixation allowed the merging of events within a coverage region that were not mergable before (2^nd^ merge). As a next step, the coverage region was split into potential gene regions (e.g. forward strand region and reverse strand region). Noise events were filtered by excluding un-spliced events which were incompatible with spliced events (unspliced events extending across an exon-intron-boundary without having an alternative splice site), internal unspliced events (events that lie within introns of other events and do not overlap with any other event) and external events without spliced reads and a coverage below 25 % of the highest coverage in the gene region. These filters excluded spurious expression within introns as well as upstream and downstream of the event in the region of coverage. After a third merge, transcript annotations were predicted and genes identified. As a result, 6,415 putative genes were identified. A summary of the transcript discovery data is presented in Table 1. The length, genomic location and sequence coverage of all putative genes identified are described in Additional file 5. Due to the lack of an annotated reference genome and the fragmentation of our *I. ricinus* genome, a full genome annotation is hardly possible at this stage and will be subject of further investigations. Whole genome annotation is even further undermined by the fact, that a large part of the *I. ricinus* amino acid sequences registered at NCBI are putative and accordingly require individual detailed analyzes. Nevertheless, the genomic information provided including this first list of putative genes contributes to the identification of so far unknown genes of *I. ricinus*. It will allow to further analyze splice site usage, to identify tick specific splice sites, average gene lengths etc., thereby further elucidating the genetic structure of the main European tick.

**Table 1:** Summary of transcript discovery analysis for identification of putative genes in the *I. ricinus* genome

Transcript discovery analysis^*a*^	
Number of genes identified	6,415
Length of the genes	250 – 30,734 bp
Number of transcripts/gene	1 – 16
Length of longest transcript/gene	75 – 5,352 bp
Number of reads/gene	10 – 15,986
Number of spliced reads/gene	1 – 10,518

^*a*^Detailed information for all 6,415 identified putative genes is included in Additional file 5

### Transcriptome sequencing and *de novo* assembly

*De novo* assembly of the mRNA reads was performed on 22,125,356 quality trimmed reads generating 60,693 contigs with a minimum size of 200 bp. The assembled contigs as well as the raw sequencing reads are publicly available at NCBI (GCJO01000000 and SRP051469, respectively). The assembled contigs were aligned against 16,002 contigs available from a recently published mixed midgut and salivary gland transcriptome assembly of partially fed *I. ricinus* ticks [19]. Instead of a mixed approach, we focused on the midgut only and used naïve *I. ricinus* ticks. Although we retrieved only around one third of the total midgut read numbers of the mixed approach and our reads were only around one third of the length, we were able to enlarge the total transcriptome lengths (lengths spanned by contig sequences) by 11 Mb to 25.5 Mb and identified 1,144 additional transcriptome sequences. Moreover, we could map our contigs back to 96 % of the coding sequences from the above combined assembly of mRNA sequences from midgut and salivary glands. This large overlap is probably predominantly due to ubiquitously expressed house-keeping genes. The high transcriptome overlap between unfed and partially fed stages of the midgut is also in line with the 85 % overlap (across all time points) of proteins expressed in the midgut and salivary glands of partially fed *I. ricinus* ticks reported by Schwarz et al. [19] and Kotsyfakis et al. [20], which was also suggested to derive from ubiquitously expressed house-keeping genes. The large overlap between the two completely different approaches is a strong indication of a virtually complete coverage at least of the most abundant *I. ricinus* midgut transcripts in unfed and partially fed stages as well as a complete overall transcript coverage of house-keeping transcripts.

### Annotation of the *I. ricinus* midgut transcriptome

In the following, we combined an alignment-based strategy with a motif search-based strategy for annotating transcripts of the unfed midgut of *I. ricinus* tick.

**Fig. 1:**
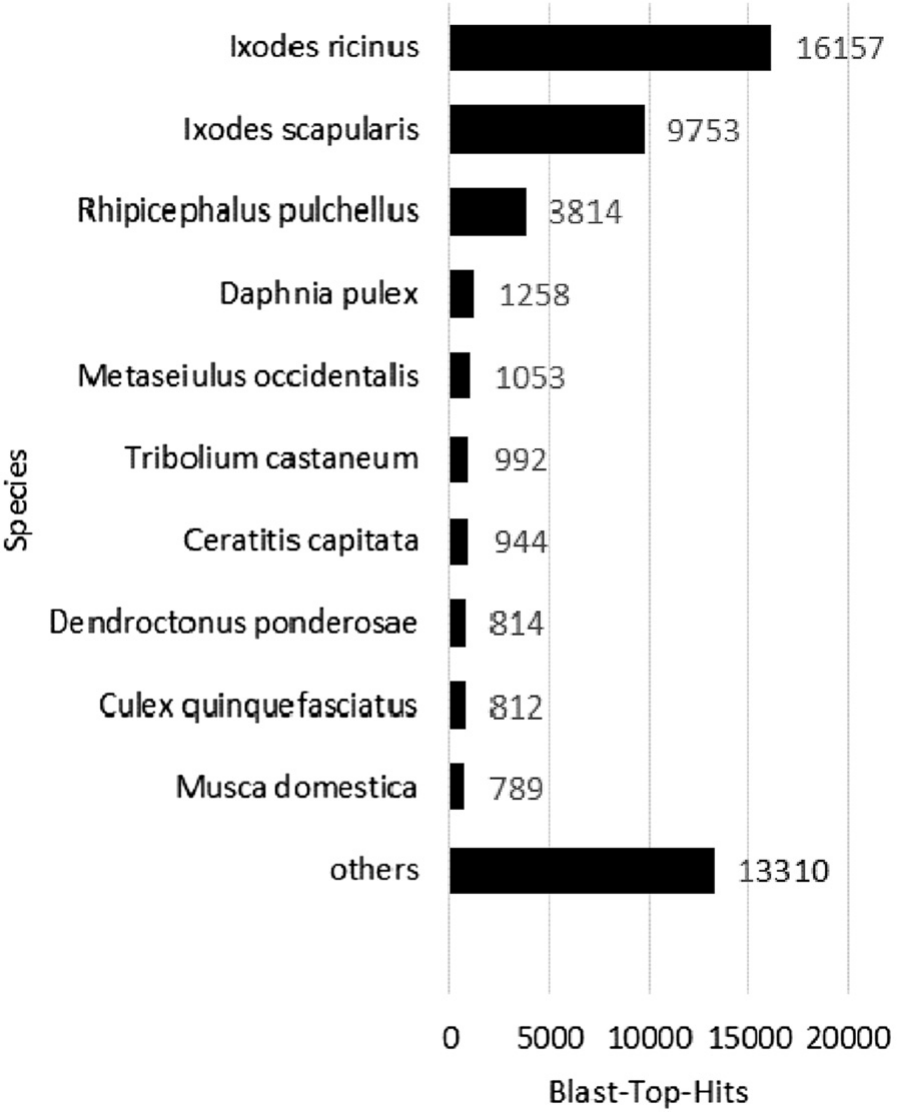
Species ranking. Species are ranked by number of transcriptome contigs with a top hit in the Blast search against NCBI Arthropoda database

All 60,693 assembled transcriptome contigs were blasted against all arthropod protein sequences available at NCBI at the time of analysis. For 49,707 contigs, a Blast result matching the evidence filter criteria was retrieved. Analysis of species distribution revealed that 16,157 contigs had an *I. ricinus* protein as best match and 9,753 contigs showed highest similarity to a protein identified in *I. scapularis*. These two species had the highest top-hit numbers (Fig. 1). This reflects well the close relationship between these two species and validates the quality of our assembly. In total, 36,386 contigs matched best to proteins of the 10 most prevalent top-hit species, out of which 29,724 contigs (82 %) matched best to a protein sequence derived from a tick species (Fig. 1).

Thirteen thousand, nine hundred ninety-five annotations from GO database for the three classes “molecular function”, “biological process” and “cellular component” could be mapped to 11,665 contigs out of the 49,707 contigs with Blast result. Out of these 11,665 contigs with annotations in the different classes, 4,826 contigs (41 %) passed the high evidence criteria described in the Methods Section.

Since sequence information and even more functional or localization information are very limited for ticks in general and *I. ricinus* in particular, valid annotation is very scarce. To overcome database limitations, our analysis pipeline was expanded to include motif search by InterProScan. With this protein function classification approach, 12,731 additional GO annotations could be retrieved. Merging the two annotation strategies yielded 26,726 validated annotations. We further expanded the mapping by inserting a second GO layer that added “biological process” and “cellular component” annotations to transcripts based on their molecular functions (ANNEX analysis). This added another 4,108 new annotations and 531 annotations were corrected. In addition, this approach normalized variations in annotation practice of public databases as each molecular function term became always annotated beyond that to its biological process and cellular component; making the annotation more consistent and more robust.

**Fig. 2:**
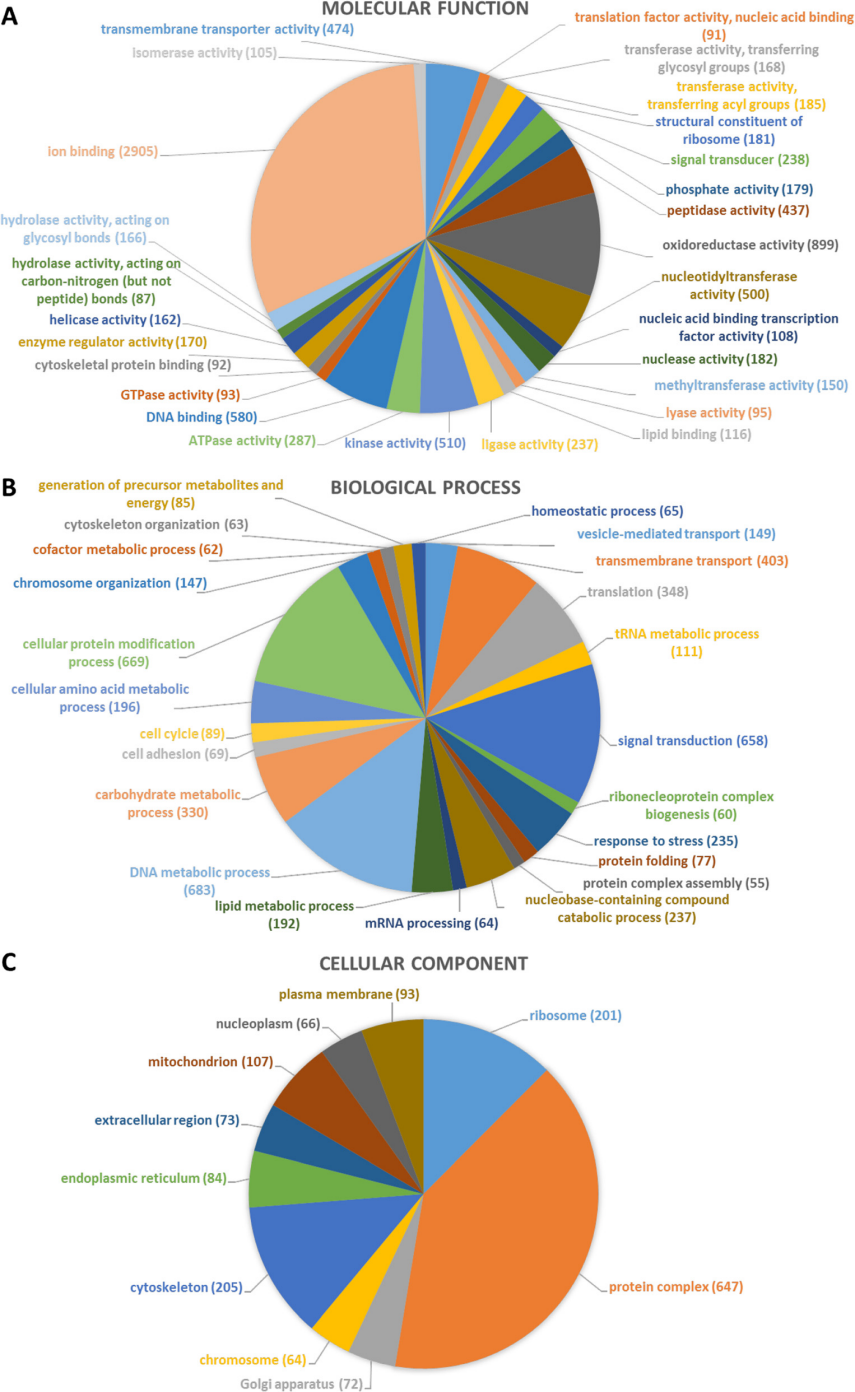
Multilevel distribution of annotated mRNA sequences by GO category. Distribution is shown for the GO categories “molecular function” (**a**), “biological process” (**b**) and “cellular component” (**c**). Only terms with a minimum of 50 annotated sequences are shown. Detailed information about the number of assigned sequences per term are included in Additional Files 6 and 9. The numbers in brackets represent the number of sequences that are annotated to this term

Ten thousand, four hundred eighty-seven sequences (17 %) were annotated with high evidence for molecular function, biological process and/or cellular component by 30,843 GO entries. A mapping result not matching the evidence filter for annotation was found in 11 % of sequences. Fifty-four percent of the contigs retrieved a Blast result for which no information was available in the GO database and for which no motif signatures were identified. This high number of sequences with Blast result, but without further annotation for function, process or cellular component, reflects the current low level of annotative information about arthropod sequences. Eighteen percent of the 60,693 contigs remained without a Blast hit with a sufficient similarity score and e-value. Here we present the most extensive transcriptome annotation for function and localization based on a combined annotation approach available for *I. ricinus* so far. All sequences and the corresponding annotations are provided as Additional file 6. GO terms to which at least 50 sequences were annotated are presented in addition in Fig. 2 for the different GO classes.

Within the GO class molecular function, “ion binding” (2,905 contigs annotated), “oxidoreductase activity” (899 contigs annotated), “DNA binding” (580 contigs annotated), “kinase activity” (510 contigs annotated) and “nucleotidyltransferase activity” (500 contigs annotated) were the five most important molecular functions among all sequences, when several molecular functions (e.g. “DNA binding” and “transcription factor activity") were allowed to be assigned to the same contig. When all 4,168 sequences annotated for “catalytic activities” were combined, they represented together with the sequences annotated for “binding activity” (4,027 sequences) the most diverse subsets within the molecular function group. They were followed by the “transporter activity” subset which was represented by only 498 sequences. Since the catalytic activity is the most prevalent and the most heterogeneous group among all molecular function groups, it was further analyzed in more detail.

In addition to the combined annotation approach by alignment and motif search, enzyme code mapping classified 2,356 contigs into the different enzyme classes. Table 2 presents the variety of the different enzyme classes in the naïve *I. ricinus* midgut. The most abundant enzymes were classified as hydrolases with 810 assigned contigs. In parallel, 1,594 sequences showed “hydrolytic activity” according to the combined annotation. In summary, the order of enzyme classes according to the number of assigned sequences was identical in both annotation approaches. The total numbers of sequences assigned to the different enzyme classes were always higher for the combined annotation approach. The reason is, that sequences can be annotated according to their motif signature (e.g. as hydrolases) without being annotated as such in the GO database - either because the function has not been annotated to this sequence yet or because the sequence itself has been unknown so far. This is once more a reflection of the limited annotative information for *I. ricinus* and highlights the need to combine different annotation strategies for non-model species. Moreover, the identical order in assigned enzyme classes for the combined approach and the enzyme code mapping confirms the uniform identification of motifs without prioritizing or shifting overall annotations.

**Table 2:** Enzyme Code (EC) distribution in the naïve *I. ricinus* midgut

EC classes	EC mapping (# of sequences)	Combined annotation^*a*^(# of sequences)
Hydrolases	810	1,594
Transferases	777	1,043
Oxidoreductases	454	899
Ligases	179	237
Isomerases	71	105
Lyases	65	95

^*a*^Combined alignment and motif-search annotation

In the category biological processes, metabolic processes involving DNA had the highest diversity in mRNA expression (683 different sequences). These processes include e.g. DNA replication and methylation as well as transcription. Six hundred sixty-nine different sequences coded for proteins involved in protein modification, including pre-, co- and post-translational modifications. “Signal transduction” and “transmembrane transport” were also among the most diverse processes in the naïve midgut with 658 and 403 annotated sequences, respectively. 40 % of mRNA transcripts assigned to cellular components (647 sequences) code for proteins that are part of macromolecular complexes of proteins covalently bound to molecules such as nucleotides, metal ions or lipid anchors. 205 distinct mRNAs were assigned to the cytoskeleton. Translational activity is represented by 201 different mRNA transcripts coding for proteins that are part of the ribosomal complex.

Thus, the high level of transcripts in the naïve midgut related to DNA processing, such as transcription (683 sequences), translation (201 sequences) and the protein modification machinery (669 sequences) vouch for a very active protein metabolism already in the unfed stage of the tick.

The summary of the biological processes in the naïve midgut (Additional file 7) shows that “metabolic and cellular processes” were among the most prevalent ones, which aligns with our finding that “catalytic activity” is the most frequent molecular function. Systems analysis further suggests that the midgut is in a metabolic phase in the unfed tick to recover the energy spend for questing. Moderate numbers of transcripts were implicated in the processes “response to stimulus”, “biological regulation” and “signaling”, apparently in an effort to prepare the midgut for an anticipated blood meal that would activate changes in biological regulation and signaling. Complementary to these biological processes, the encoded molecular functions “transporter activity” and “enzyme regulator activity” as well as “protein and nucleic acid binding transcription factor activity” seem to be on hair-trigger alert to enable the tick midgut to shift rapidly from a catabolic to an anabolic metabolism by adapting enzyme activities and transcription. In contrast, transcripts coding for proteins involved in “developmental processes” or “locomotion” were rare. At later feeding stages, these processes may become more important due to structural growth of the midgut during feeding and in particular during molting of the tick, not only in the midgut but also in other organs. The “catabolic-while-ready-to-sensing-status” of the tick is further supported by the preferred cellular localization of encoded proteins in protein complexes or cellular organelles, such as e.g. mitochondria. As the “power plants” of the cells, activity in these organelles aligns and supports the energy recovering mode. On the other hand, numbers of encoded proteins associated with the membrane or extracellular regions were relatively low at the unfed stage. Expression of these proteins may increase only when the midgut undergoes dramatic expansion during the late feeding stage.

Considering only annotations of transcripts that were not found by Schwarz et al. [19] in neither the midgut nor the salivary glands of fed *I. ricinus* ticks, the patterns are similar to the overall transcript annotations (Additional file 6). As Schwarz et al. [19] presented a mixed transcriptome assembly of midgut and salivary glands of partially fed *I. ricinus* ticks, a more detailed comparison of the partially fed to the unfed midgut is not possible.

### Annotation of the *I. ricinus* midgut proteome

This study provides the first insight into the midgut proteome of unfed *I. ricinus* ticks.

After separation of midgut proteins according to size and isoelectric point, 1,161 protein spots were picked, digested and analyzed by mass spectrometry. In a combined search, PMF and up to 10 MS/MS spectra per protein spot were searched via Mascot against the assembled mRNA sequences from our midgut transcriptome (Additional file 8). 574 gel spots were significantly identified by mass spectrometry and among these, 285 distinct proteins expressed in the naïve midgut could be annotated for function and/or localization. All mass spectrometry data are available via ProteomeXchange (PXD001796).

**Fig. 3:**
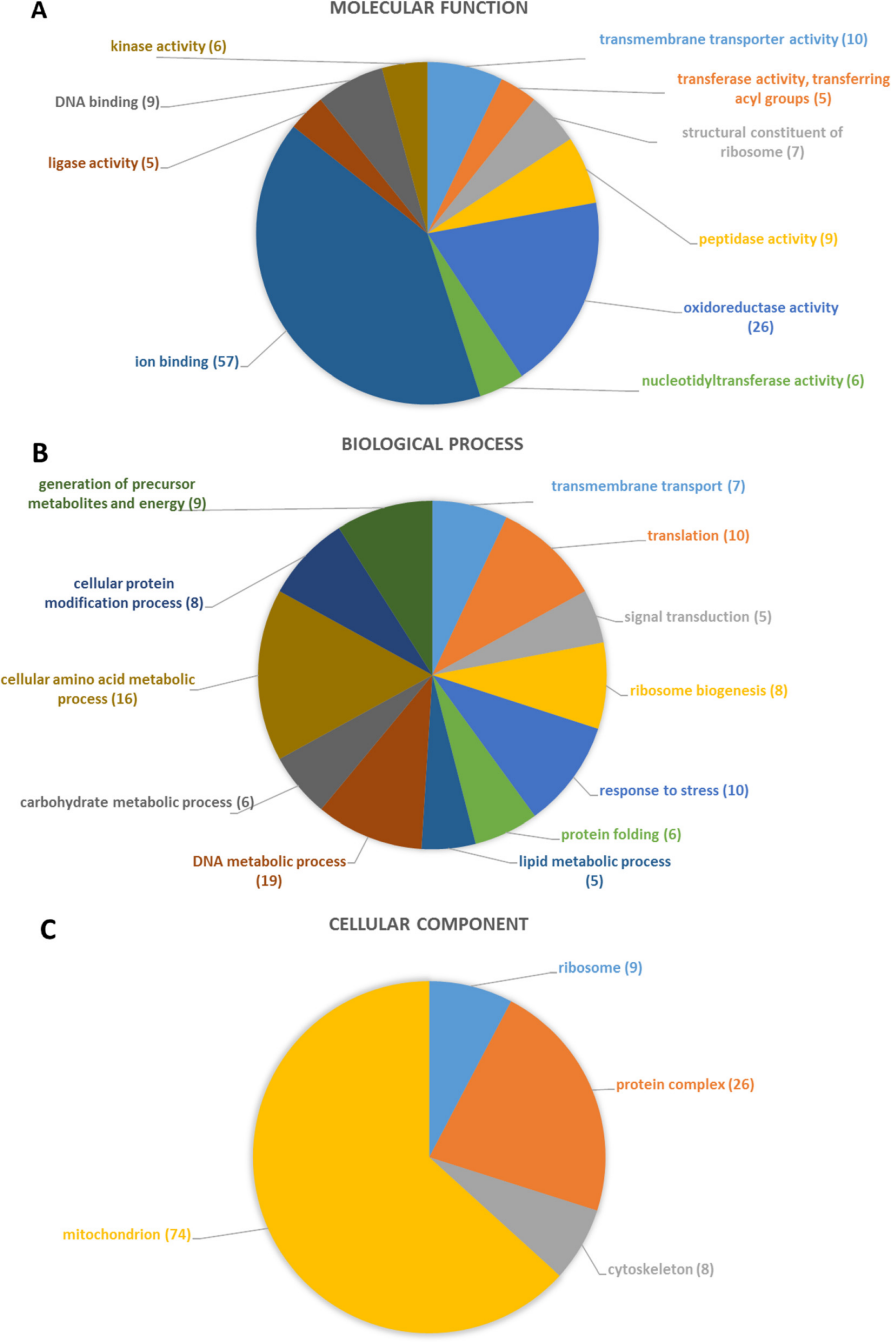
Multilevel distribution of annotated proteins by GO categories. Distribution is shown for the GO categories “molecular function” (**a**), “biological process” (**b**) and “cellular component” (**c**). Only terms with a minimum of 5 annotated sequences are shown. Detailed information about the number of assigned sequences per term are included in Additional Files 8 and 9. The numbers in brackets represent the number of sequences that are annotated to this term

In order to cross-validate our transcriptome annotations with the protein annotations, Blast hits retrieved for mRNA contigs matching a protein in the above Mascot search (i.e. against our in-house transcriptome database) were compared to the hits of the Mascot search obtained for this protein against public arthropod protein sequence databases. In total, a significant hit for both Mascot searches was retrieved for 427 gel spots. Reasons for low scores in one of the Mascot searches include mainly limitations of the Arthropoda database and/or a very low expression of the protein and poor MS spectra. Combining the different isoforms of a protein, these 427 gel spots correspond to 166 distinct proteins. For 154 proteins (93 %) of the above 166 proteins with matches in both searches (i.e. our transcriptome and the public arthropod proteins), annotations were identical. When the annotations were extended to similar results found in Mascot and Blast, i.e. same protein family or same protein, but different species, 98 % of protein identifications (162 out of 166) overlapped. This overlap unequivocally links these mRNAs and proteins, and furthermore confirms by cross-validation the robustness of the annotation results of both, the transcriptome and the proteome, and the annotation strategies used. In addition to the above 166 proteins, 117 proteins with a significant score in the Arthropoda Mascot search only and 2 other proteins with a significant score in the internal mRNA database search only were identified, albeit without cross-validation. There are obvious overlaps between transcriptome and proteome annotations (Figs. 2 and 3). The most important molecular functions are in both cases “catalytic activity” and “ion binding”. Most proteins are found in the mitochondria, which further supports our observations from the transcriptome of a catabolic metabolism in the unfed midgut during questing. The biological process with the highest number of assigned transcripts, “DNA metabolic processes”, shows highest diversity in the proteome as well. Proteins involved in the “generation of precursor metabolites and energy” are also among the most prevalent categories further highlighting the catabolic profile of the unfed midgut. Altogether, there were only small differences between the annotation profiles of the transcriptome and the proteome in the unfed midgut and overall annotation patterns were very similar. Since the unfed midgut is in a steady state, similar annotation profiles for transcriptome and proteome are absolutely plausible. One would expect to see a difference between transcriptome and proteome annotations with the start of the blood meal triggering changes in the metabolism. This change would be initiated by a shift in the transcription profile, followed by a delayed change in protein expression profiles. A comprehensive overview of the annotation patterns of the transcriptome and the proteome is shown in Figs. 2 and 3 and Additional file 9. A detailed list of the annotations for each protein spot and each mRNA sequence is provided as Additional files 6 and 8.

In summary, 285 distinct proteins expressed in the naïve midgut were significantly identified and furthermore annotated for their function and/or cellular localization. An annotated 2D gel picture and a table summarizing all Blast and Mascot annotations are included as Additional files 10 and 8, respectively. These coding sequences and annotations contribute in an essential manner to the elucidation of the *I. ricinus* proteome and will facilitate investigation of tick-pathogen and tick-host interactions as well as identification of potential vaccine candidates.

## Conclusions

We constructed here a first reference genome comprising an estimated two thirds of the unique sequences of the *I. ricinus* tick, the most important European disease vector. When comparing our genome contigs to *I. scapularis* as a reference genome, 89 % of the *I. scapularis* scaffolds were found to be homologous to sequences of our *I. ricinus* assembly. In addition, more than 6,000 putative new genes were identified. Although not complete, the genomic information provided contributes to the identification of so far unknown genes of *I. ricinus* and provides a robust basis for further elucidating the complete genetic structure of this main European tick.

These genomic data were complemented by about 60,000 assembled transcriptome sequences and, even more important, by the first large scale functional and local annotation corresponding to more than 10,000 transcripts and 285 proteins expressed in the naïve midgut. It will be the scope of future studies to confirm their predicted functions and cellular localizations.

The combination of both, transcriptome and proteome annotations in the GO categories “molecular function”, “biological process” and “cellular component”, provides a systems overview of pathways and metabolic activity in the unfed *I. ricinus* midgut. Our data provide the basis for investigating specific pathways in the tick as well as tick-pathogen and tick-host interactions. They also allow to develop immunoprophylactic approaches by reverse vaccinology and other mitigation strategies against the most important European disease vector. The genome as a reference allows furthermore to identify gene categories, e.g. immunity-related genes, by homology search. In summary, the presented databases will accelerate investigations of tick-host and tick-pathogen interactions, identification of vaccine candidates based on concealed antigens and studies of midgut functions such as blood digestion.

## Methods

### Tick dissection

Pathogen-free *I. ricinus* ticks were purchased from Charles River Laboratories, Ireland. The colony was initially built up from *I. ricinus* adults collected in Ireland, Slovakia and Germany.

Prior to extraction or dissection, ticks were first washed in 0.1 % benzalkonium chloride for 5 min, second in 70 % ethanol for 1 min and finally rinsed twice with sterile distilled water. High molecular weight genomic DNA was extracted from whole unfed male adults, while RNA and proteins were obtained from the midguts of unfed female adults. Immediately prior to dissection, ticks were fixed dorsal-side up in a paraffin layer by melting the paraffin locally and inserting the legs and ventral side into the paraffin. Tick were covered with either RNase inhibitor solution (RNAlater, life technologies, Carlsbad, CA, USA) or protease inhibitor solution (Pierce Protease Inhibitor Tablets, Thermo Fisher Scientific Inc., Waltham, MA, USA). Dissection was performed on a cool pack. Explanted midguts were immediately transferred into RNase/protease inhibitor solution and kept on ice until storage at -80 °C.

### Genomics and Transcriptomics

#### a. High Molecular Weight Genomic DNA extraction

High molecular weight DNA was extracted according to a modified protocol from Hill and Gutierrez [21]. In brief, 6 male ticks were frozen in liquid nitrogen and ground with a pestle. Powder was resuspended in 20 volumes of extraction buffer (10 mM Tris-Cl, pH 8.0, 100 mM EDTA and 1 % SDS) and incubated for 30 min at 37 °C under gentle mixing. After centrifugation at 4,000 g for 10 min, proteinase K was added to the supernatant to a final concentration of 50 μg/ml and incubated at 37 °C overnight under gentle mixing. RNase cocktail (0.05 units RNase A and 2 units RNase T1 per mg tick tissue) was added and incubated at 50 °C for 3 h. DNA was extracted using one volume 25:24:1 phenol:chloroform:isoamylalcohol mix and centrifuged for 5 min at 10,000 g and 4 °C. The aqueous phase was transferred to a fresh tube and the extraction repeated. A final extraction was done with 24:1 chloroform:isoamylalcohol. Extracted DNA was precipitated by adding 1/10 volume of 3 M sodium acetate (pH 5.5) and 2 volumes of 100 % ethanol and centrifugation for 10 min and 4 °C at 19,000 g. The pellet was washed with 70 % ice-cold ethanol, dried at room temperature and resuspended in 150 μl TE buffer (10 mM Tris, 1 mM EDTA). DNA was quantified with the Quant-iT Picogreen dsDNA Assay kit (Life technologies, Carlsbad, CA, USA) and integrity of high molecular weight DNA was confirmed by agarose gel electrophoresis.

#### b. Messenger RNA (mRNA) extraction

One hundred twenty *I. ricinus* midguts were homogenized in a dounce homogenizer. No technical replicates could be performed due to cost restraints, so a potential bias introduced by sample preparation and/or during the detection/analysis workflow cannot be totally excluded and may skew the results. While this may represent a partial limitation of the study, the biological variation has been reduced by pooling individuals for mRNA extraction, library preparation and sequencing. Total RNA was extracted with TRIzol Reagent (Life technologies, Carlsbad, CA, USA) and DNA was digested with DNAfree kit (Life technologies, Carlsbad, CA, USA) according to the manufacturer’s protocol. RNA was purified by use of RNAcleanXP beads (BeckmanCoulter, Brea, CA, USA) with a sample to beads ratio of 1:1.8. mRNA was extracted with Dynabeads RNA DIRECT Micro Kit (Life technologies, Carlsbad, CA, USA).

#### c. Library preparation and sequencing

For genomic DNA, a paired-end library was prepared according to the manufacturer’s paired-end sample preparation guide (Illumina, San Diego, CA, USA) and run on an Illumina HiSeq™ 2500 sequencer (Illumina, San Diego, CA, USA) by Ambry Genetics (Aliso Viejo, CA, USA). Initial data processing and base calling of 100 base pair (bp) reads was done with RTA 1.17.20 (HiSeq Control Software 2.0.5, Illumina, San Diego, CA, USA) and sequence quality filtering script was executed in the Illumina CASAVA software (V1.8.2, Illumina, San Diego, CA, USA) by Ambry Genetics (Aliso Viejo, CA, USA).

*I. ricinus* midgut whole transcriptome library was prepared with the Ion Total RNA-Seq Kit v2 (life technologies, Carlsbad, CA, USA) according to the manufacturer’s protocol. Sequencing was accomplished on an Ion PGM™ sequencer with the Ion PGM™ Template OT2 200 Kit for template preparation and the Ion PGM™ Sequencing 200 Kit v2 for sequencing on Ion 314™ Chips v2 and Ion 318™ Chips (life technologies, Carlsbad, CA, USA) according to the manufacturer’s protocol.

#### d. *De novo* assembly

All *de novo* assemblies were accomplished in CLC Genomics Workbench 7.4 (CLCBio, Aarhus, Denmark). As described recently, CLCbio and other De-Bruijn graph based algorithms like e.g. ABySS and Velvet are well suited for short paired-end reads assemblies and perform comparably [22]. CLCbio was chosen due to its better resource management (especially for memory capacity). In addition, the multiple k-mers based assembly by CLCbio, as it was used for the transcriptome *de novo* assembly, performed very well in comparison to other assemblers, (namely MIRA, Newbler, SOAPdenovo, Trinity and Velvet-Oases) [23].

Genomic Illumina paired-end reads had a length of 100 bp and were not further trimmed before *de novo* assembly because of the high accuracy in base calling of the Illumina HiSeq™ 2500 leading to a phred score above 30 for most bases. In this case, the quality increase due to trimming is low and does not affect assembly accuracy, while the number of broken pairs is increased, which may compromise assembly accuracy. Genomic sequences were assembled *de novo* using a word size of 32 and a bubble size of 80, as recommended for the assembly of large genomes (White paper on de novo assembly in CLC Assembly Cell 4.0, CLCBio, Aarhus, Denmark). Minimum contig length was set to 1 kilobase (Kb); mismatch costs to 2; and insertion and deletion costs to 3. In order to check and to improve the quality of the assembly, paired-end reads were mapped back to the assembled contigs. For the back-mapping, the identical mismatch, insertion and deletion costs were used as for the *de novo* assembly (2, 3 and 3, respectively); the length fraction was set to 0.5 and the similarity fraction to 0.8. Thereby, local sequence errors (e.g. short insertions or deletions) as well as structural errors (e.g. scaffolding errors) were identified by mismatching reads and contigs were corrected by CLC Genomics Workbench 7.4 (CLCBio, Aarhus, Denmark), if necessary. The assembled genome contigs were analyzed for microbial contamination by alignment against the NCBI bacterial and viral nucleic acid database (04/14/2014) and sequences derived from contaminating microorganisms were removed (CLC Genomics Workbench 7.4, CLCBio, Aarhus, Denmark). An additional check of (microbial) contamination was automatically done by NCBI when uploading the contig sequences into the NCBI database.

Because of the fragmentation of the assembled genome, RNA reads were *de novo* assembled to allow the identification of transcripts for which there is only an incomplete or fragmented reference in our genome contigs. Ion PGM™ derived reads from midgut transcriptome library had a mean length of 118 bp and were depleted of remaining rRNA sequences by reference mapping (CLC Genomics Workbench 7.4, CLCBio, Aarhus, Denmark) against known rRNA sequences from Ixodes ticks (GenBank: S65924, L34292, Z74479, GU001677 and JF703110) and quality trimmed with FASTX-Toolkit [24]. The first 10 bases and all bases beyond base 300 were removed for all sequences because of a loss of quality. Only sequences with a phred score of 20 over at least 90 % of bases were kept.

While originally high phred scores were implemented, more recent publications are concerned that quality trimming could lead to missing annotations and incomplete transcript reconstruction [25]. A phred score of 20, which corresponds to a base call accuracy of 99 %, ensures a high accuracy of the assembled transcripts while keeping the effect of incomplete transcript reconstruction at a minimum.

The *de novo* assembly of midgut transcriptomic sequences was performed as described above for mismatch, insertion and deletion costs and correction of contigs. However, due to the expected differences in transcript sizes, word and bubble size were calculated automatically per contig during assembly. The minimum contig length was set to 200 bases.

#### e. Redundancy analysis, homology comparisons and mapping against *I. scapularis* scaffolds and *I. ricinus* genes

The assembled contigs were analyzed for their redundancy with a duplicate removal tool. The contigs were filtered for sequences which share a common sequence of at least 20 bases and an alignment score above 80 % of the optimal score in the rest of the sequence. The optimal score is defined as the score a sequence would get if it aligned perfectly to the consensus for a group of duplicates.

The assembled genome contigs were analyzed for their homology against *Dermatophagoides farinae*, *I. scapularis*, *Metaseiulus occidentalis and Tetranychus urticae* genome scaffolds as well as *Rhipicephalus microplus* and *Varroa destructor* genome contigs [NCBI Genbank: ASGP00000000.1, ABJB000000000.1, AFFJ00000000.1, CAEY00000000.1, ADMZ00000000.2 and ADDG00000000.1] by local blastn. Match/mismatch costs were set to 1 and 2, gap costs to 5 for existence and 2 for extension of a gap. The word size was set to 36 and the expectation value to 10.

Mapping of the assembled genome contigs against public *I. scapularis* genome scaffolds [NCBI Genbank: ABJB000000000.1] was performed with the same mismatch and insertion/deletion costs as for the assembly. Mapping criteria were set to a length fraction of 0.5 and a similarity fraction of 0.8 (at least 50 % of bases need to align to the reference with an overall identity of minimum 80 %).

All *I. ricinus* genes in the NCBI Genbank at the time of analysis (date: 03/03/2015, 849 genes total) were searched in a blastn analysis against our assembled contigs with match/mismatch cost of 2/3 and gap costs of 5/2 (existence/extension). The word size was set to 11 and the expectation value to 10.

Mapping, local blastn and duplicate removal analyses were performed with CLC Genomics Workbench 7.4 (CLCBio, Aarhus, Denmark).

#### f. Identification of putative genes in genome contigs

With a large gap read mapper, mRNA sequences were mapped to the assembled genome contigs. The large gap mapper used is based on the standard read mapper of the CLC Genomics Workbench. Maximum number of hits was set to 10 and maximum distance from seed segment to 50 Kb. Large gap read mapping was used for transcript discovery including the following splice sites: exon GT intron AG exon, exon GC intron AG exon, exon GC intron TG exon, exon GC intron AA exon, exon GC intron GG exon, exon GT intron TG exon, exon GT intron AA exon, exon AT intron AC exon, exon AT intron AA exon, exon AT intron AG exon, exon AT intron AT exon, automatic noise filtering and prediction of open reading frames with a minimum length of 100 bases. Large gap read mapping and transcript discovery were performed in CLC Genomics Workbench 7.0.4 (CLCBio, Aarhus, Denmark).

#### g. Mapping of RNA reads

All mappings were performed in CLC Genomics Workbench 7.0.4 (CLCBio, Aarhus, Denmark).

Quality trimmed reads from RNA sequencing were mapped against the assembled contigs from genome sequencing, which thus served as reference. The same mismatch and insertion/deletion costs were used as for the assembly. Mapping criteria were set to a length fraction of 0.5 and a similarity fraction of 0.8.

In a blastn based alignment approach, transcriptomic contig sequences were mapped against publicly available contigs from a mixed *I. ricinus* salivary gland and partially fed midgut assembly [19] with match/mismatch cost of 2/3 and gap costs of 5/2 (existence/extension). The word size was set to 11 and the expectation value to 10.

#### h. Annotation of transcriptome contigs

Assembled transcriptome contigs were annotated via the Blast2GO [26] software plugin (Genomics of Gene Expression, Valencia, Spain) for CLC Workbench 7.0.4 (CLCBio, Aarhus, Denmark).

In brief, sequences were blasted locally by blastx against the NCBI Arthropoda protein database (date of database: 04/14/2014) and Gene Ontology (GO) IDs were mapped. Out of the mapped GO IDs, those that passed an annotation cut-off of 55 for the lowest term per branch and a GO-weight of at least 5 were annotated to the contigs. In addition, annotation was restricted to Blast hits passing an evidence filter of a minimum e-value of 1xe^−6^ and a similarity of at least 30 % throughout the alignment length.

In a second approach, assembled transcriptome contig sequences were analyzed with InterProScan [27] (default parameters). InterProScan uses several prediction algorithms and combines their individual results. Protein family membership prediction and presence of functional domains and sites are combined to profile protein sequences. The results of both annotation approaches, based on alignment by blast and motif search by InterProScan, were merged.

A second GO layer was inserted that linked and, whenever necessary, corrected annotations for molecular function with annotations for biological processes, the mRNA sequences are involved in, and annotations for cellular components, they are active in, by performing ANNEX analysis [28]. Finally, GO-Slim algorithm was run to summarize GO annotations to a general functional scheme. The transcriptome annotation pipeline is illustrated in Additional file 11.

#### i. Visualization of transcriptome contig annotation

In order to visualize the distribution of GO terms annotated to transcriptome contigs, direct acyclic graphs (DAG) for molecular function, biological process and cellular component were built for the annotated sequences with the Blast2GO [26] software plugin (Genomics of Gene Expression, Valencia, Spain) for CLC Workbench 7.0.4 (CLCBio, Aarhus, Denmark). Since the annotation results of all sequences were joined within the same DAG, it was called combined DAG. A node score filter of 12.0 and a sequence filter of 10 was set. The combined DAGs for the GO categories “molecular function”, “biological process” and “cellular component” are shown in Additional file 12. Out of the combined DAGs, pie charts were created with a sequence filter of 50 for the multilevel charts and 1 for the level charts.

### Proteomics

#### a. Protein extraction

One hundred twenty dissected midguts were pooled for protein extraction. As no technical replicates could be performed due to cost restraints, we cannot exclude that sample preparation and/or the detection/analysis workflow may skew the results, representing a partial limitation of the study. However, the biological variation has been reduced by pooling individuals for protein extraction and mass spectrometry analysis. The midguts were pelleted by centrifugation at 3,000 g for 5 min at 4 °C and washed in phosphate buffered saline (1.37 M NaCl, 27 mM KCl, 100 mM Na_2_HPO_4_, 18 mM KH_2_PO_4_). 1 mL lysis buffer (30 mM Tris-Cl, 7 M Urea, 2 M Thiourea, 4 % CHAPS, pH 8.5) supplemented with protease inhibitor (Pierce protease Inhibitor Tablets, Thermo Fisher Scientific Inc., Waltham, MA, USA) was added and suspension homogenized with a dounce potter. The homogenate was incubated on ice for 30 min on a rocket shaker and centrifuged at 15,000 g for 20 min at 4 °C. Proteins in the supernatant were quantified with the 660 nm Protein Assay (Thermo Fisher Scientific Inc., Waltham, MA, USA) and precipitated by chloroform/methanol using 4 volumes of 100 % methanol, 1 volume of chloroform and 3 volumes of double-distilled water and centrifuged at 5,000 g for 5 min. The aqueous layer was removed and 4 volumes of 100 % methanol added. After centrifugation at 14,000 g for 30 min, any liquid was removed and the pellet was air-dried before being resuspended to a final concentration of 5 mg/mL in lysis buffer.

#### b. 2D-GE-MS/MS

Extracted proteins were labeled with CyDye2 (GE Healthcare Ltd, Chalfont St Giles, GB) according to the manufacturer’s protocol. Isoelectric focusing was done on a 24 cm ImmobilineDryStrip, pH 3-10 nonlinear (Pharmacia Biotech, Uppsala, Sweden) in an Ettan™ IPGphor3 (GE Healthcare Ltd, Chalfont St Giles, GB) with a maximum of 10,000 V and a total of 110,500 Vh. The second dimension separation was performed on a 12.5 % (v/v) ExcelGel polyacrylamide gel in an EttanDALTsix electrophoresis unit (GE Healthcare Ltd, Chalfont St Giles, GB). The gel was scanned with a Typhoon FLA 9500 (GE Healthcare Ltd, Chalfont St Giles, UK) and pick lists were generated with Delta2D software (V4.2, Decodon GmbH, Greifswald, Germany). Selected spots were picked with an Ettan Spot Picker (GE Healthcare Ltd, Chalfont St Giles, GB). The picked proteins were washed twice in 50 mM Ammonium bicarbonate in 50 % Methanol (v/v), dehydrated in 75 % acetonitrile (ACN, v/v) and then dried. Trypsin digestion (Trypsin Gold, Promega, Madison, WI, USA, 5 μl/mL in 20 mM ammonium bicarbonate, incubation for 6 h at 37 °C), peptide extraction (50 % ACN (v/v)/0.1 % trifluoroacetic acid (v/v), incubation for 20 min at 37 °C) and spotting was done with an EVO 2 (Tecan, Maennedorf, Switzerland). Combined peptide mass fingerprinting (PMF) and tandem mass spectrometry (MS/MS) was done by matrix-assisted laser desorption/ionization – time of flight/time of flight (MALDI-TOF/TOF) on an AB SCIEX TOF/TOF 5800 system (AB Sciex, Framingham, MA, USA) using reflector mode.

#### c. Annotation of protein spots

Per analyzed spot, the PMF and a maximum of 10 MS/MS spectra were used for protein identification by Mascot search (V2.3, Matrix Science Ltd, London, UK) against the NCBInr database limited to the taxonomy of Arthropoda (2,341,519 sequences, download date 08/05/2014) and assembled mRNA sequences from *I. ricinus* midgut transcriptome sequencing (364,158 sequences). Peptide mass tolerance was set to ± 100 ppm, fragment mass tolerance to ± 0.5 Da with a maximum of two missed cleavages. As variable modifications, oxidation of histidine, tryptophan or methionine, dioxidation of tryptophan and tryptophan to kynurenin modification were allowed, fixed modification was set for carbamidomethylation of cysteine. The significance threshold (*p* < 0.05) was calculated automatically by Mascot for a score minimum of 76 for MS and 50 for MS/MS for searches against the Arthropoda database and 68 for MS and 41 for MS/MS for searches against the in-house transcriptome database. The score is dependent on several factors, including in particular also the number of entries in the database, against which the spectra are searched. Since the number of entries in our in-house database was lower than the number in the public Arthropoda database, a lower score was required to reach the significance threshold of *p* < 0.05. In order to set the same significance threshold for both searches, the score threshold was adjusted to the size of the database. Additionally, a false discovery rate analysis was performed on the search results. Therefore, a concatenated target-decoy database was created for the in-house transcriptome as well as the public Arthropoda database, with decoy hits created by randomizing the target sequences. Searches were repeated against both of these databases. The highest scoring hit and the cumulative FDR for each spot are presented in Additional file 13. Comparing the protein scores at 1 % FDR (70 for the transcriptome and 67 for the Arthropoda database) to the protein scores at a *p*-value of 0.05 (72 and 80, respectively), the *p* < 0.05 threshold was stricter than the 1 % FDR threshold. Therefore, this threshold was applied to categorize the search results as significant or not significant. Based on the Mascot results and annotation on the gel, spectra were checked in addition manually.

### Data access

Public disclosure of genomic sequences and transcriptomic sequences are provided on NCBI. The Whole Genome Shotgun project has been deposited at DDBJ/EMBL/GenBank under the accession JXMZ00000000. The version described in this paper is version JXMZ01000000. The raw sequence reads have been deposited under the accession SRP051465 in the Sequence Read Archive.

The Transcriptome Shotgun Assembly project has been deposited at DDBJ/EMBL/GenBank under the accession GCJO00000000. The version described in this paper is the first version, GCJO01000000. The raw sequence reads have been deposited under the accession SRP051469 in the Sequence Read Archive.

The mass spectrometry proteomics data have been deposited to the ProteomeXchange Consortium [29] via the PRIDE partner repository with the dataset identifier PXD001796 and 10.6019/PXD001796.

## Additional files

Additional file 1:
**Homology comparison to the** ***I. scapularis*** **genome.** A description of the best match including e-value, identity, bit score and hit length are listed for each contig/scaffold. (XLSX 10588 kb) Additional file 2:
**Mapping of RNA reads against genome contigs.** The total number of mapped RNA reads and the consensus length of the mapping are described for each genome contig. (XLSX 7089 kb) Additional file 3:
**Genomic sequences corresponding to known** ***I. ricinus*** **genes.** Genome contigs corresponding to known *I. ricinus* genes including search validation parameters are listed. (XLSX 48 kb) Additional file 4:
**Homology comparison to Acari genomes (except** ***I. scapularis*** **).** A description of the best match including e-value, identity, bit score and hit length of a homology comparison against all Acari genomes registered at NCBI Genbank at the time of analysis except *I. scapularis*, namely *Dermatophagoides farinae*, *Metaseiulus occidentalis*, *Rhipicephalus microplus*, *Tetranychus urticae* and *Varroa destructor*, are listed for each contig. (XLSX 1023 kb) Additional file 5:
**Putative genes identified by transcript discovery in** ***I. ricinus*** **genome.** The length of the gene, its start and end points, its strand specificity, the number of assigned transcripts, the length of the longest transcript and the number of total and spliced assigned reads are presented for each gene identified. (XLSX 418 kb) Additional file 6:
**Transcriptome annotations for the naïve** ***I. ricinus*** **midgut.** A description of the annotated sequence, e-value, mean similarity, GO IDs, enzyme codes and InterProScan results are listed for each annotated transcript. (XLSX 3707 kb) Additional file 7:
**Distribution of annotated sequences by GO categories “molecular function” (A), “biological process” (B) and “cellular component” (C) of level 2 of the combined direct acyclic graph for the annotated transcriptome sequences.** The number in brackets represent the number of sequences that are annotated to this GO term. (PDF 495 kb) Additional file 8:
**Proteome annotations for the naïve** ***I. ricinus*** **midgut.** The proteome annotations are divided in cross-validated annotations (i.e. independently retrieved annotations of the proteins and the transcripts are identical or similar) and not significant annotations, for which the Mascot scores were below the significance threshold. (XLSX 169 kb) Additional file 9:
**Multilevel distribution of annotated transcripts and proteins.** GO terms with at least 50 assigned sequences per term for the transcriptome and 5 for the proteome are listed. (XLSX 12 kb) Additional file 10:
**Annotated 2D gel picture of the naïve** ***I. ricinus*** **midgut.** The ID numbers linked to the different spots correspond to the ID numbers of the annotated proteome in Additional file 8. (PDF 1059 kb) Additional file 11:
**Transcriptome annotation pipeline.** Data flow is shown by arrows and analytical steps by capital letters. (PDF 155 kb) Additional file 12:
**Combined direct acyclic graphs for the different GO categories “molecular function” (A), “biological process” (B) and “cellular component” (C).** (PDF 90 kb) Additional file 13:
**FDR analysis of mass spectrometry search results.** Mass spectrometry search results against concatenated target-decoy databases including protein scores and cumulative FDR. (XLSX 138 kb)

## Abbreviations

s.l: Sensu lato
LB: Lyme borreliosis
NCBI: National Center for Biotechnology Information
Kb: Kilobase
GO: Gene Ontology
DAG: Direct acyclic graph
ACN: Acetonitrile
PMF: Peptide mass fingerprinting
MS/MS: Tandem mass spectrometry
MALDI-TOF/TOF: Matrix-assisted laser desorption/ionization – time of flight/time of flight
