# Supplementary material for: Integration of Ixodes ricinus genome sequencing with transcriptome and proteome annotation of the naïve midgut
Source: BMC Genomics. 2015 Oct 28;16:871. doi: 10.1186/s12864-015-1981-7 (PMC4625525; doi:10.1186/s12864-015-1981-7)
Supplement: Additional file 7: — Distribution of annotated sequences by GO categories “molecular function” (A), “biological process” (B) and “cellular component” (C) of level 2 of the combined direct acyclic graph for the annotated transcriptome sequences. The number in brackets represent the number of sequences that are annotated to this GO term. (PDF 495 kb) [file 12864_2015_1981_MOESM7_ESM.pdf]

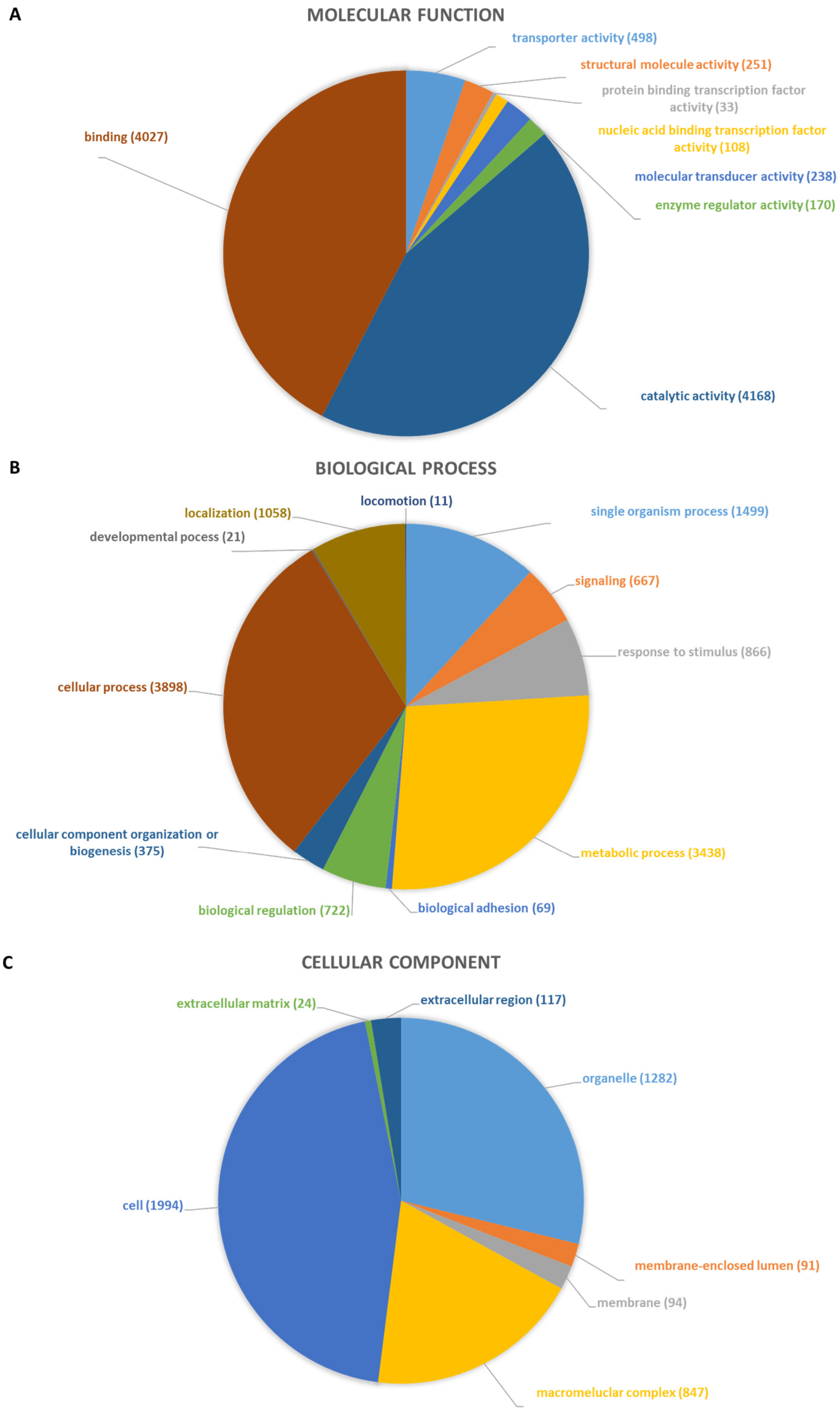

Additional file 7: Distribution of annotated mRNA sequences by the GO categories “molecular function” (A), “biological process” (B) and “cellular component” (C) of level 2 of the combined direct acyclic graph for the annotated transcriptome sequences. The numbers in brackets represent the number of sequences that are annotated to this GO term.
