# Supplementary material for: Integration of Ixodes ricinus genome sequencing with transcriptome and proteome annotation of the naïve midgut
Source: BMC Genomics. 2015 Oct 28;16:871. doi: 10.1186/s12864-015-1981-7 (PMC4625525; doi:10.1186/s12864-015-1981-7)
Supplement: Additional file 10: — Annotated 2D gel picture of the naïve I. ricinus midgut. The ID numbers linked to the different spots correspond to the ID numbers of the annotated proteome in Additional file 8. (PDF 1059 kb) [file 12864_2015_1981_MOESM10_ESM.pdf]

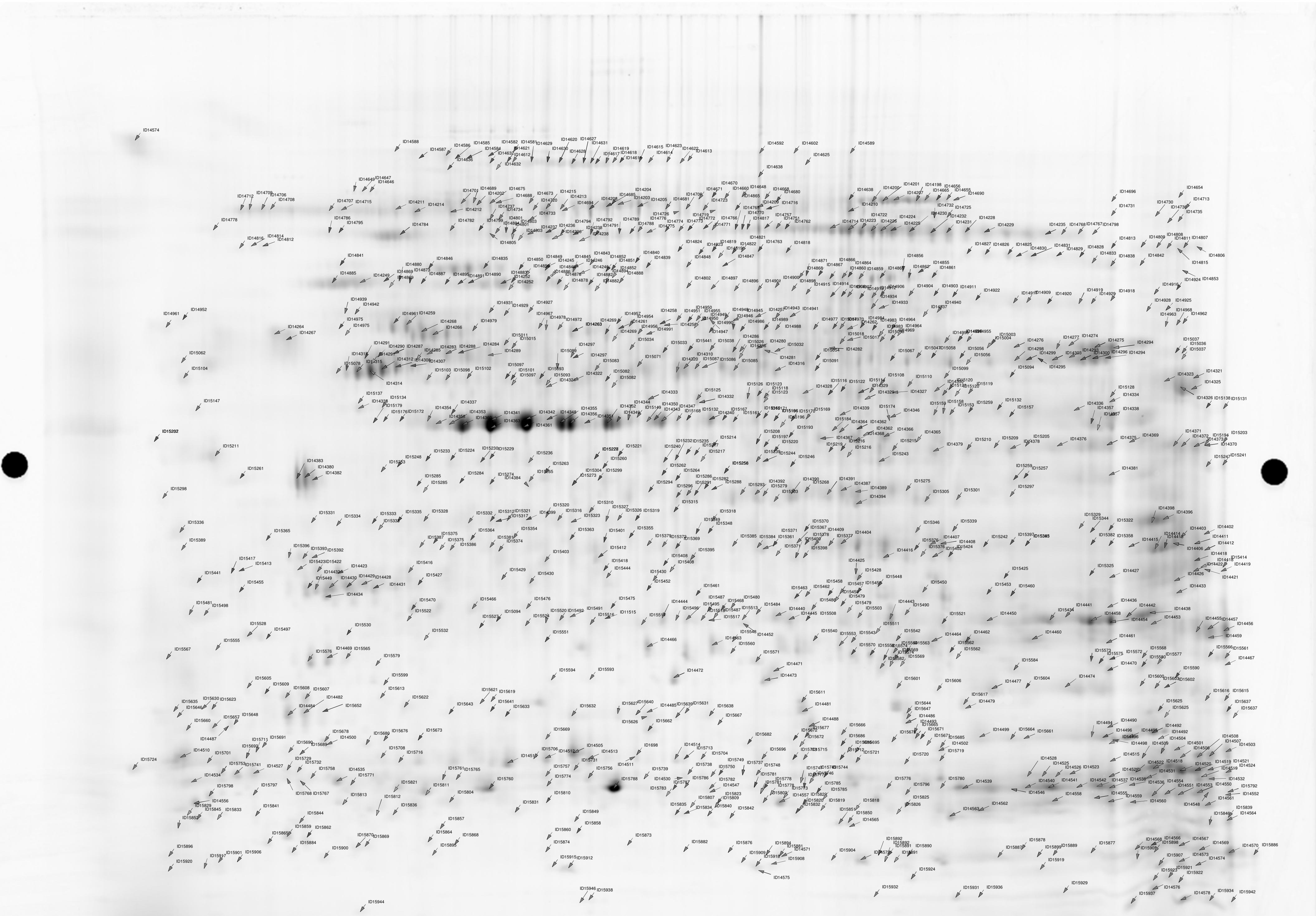

Additional file 9: Annotated 2D gel picture of the naive I. ricinus midgut. The ID numbers linked to the spots correspond to the the ID numbers of the annotated proteome in Additional file 7.
