# Supplementary material for: Integration of Ixodes ricinus genome sequencing with transcriptome and proteome annotation of the naïve midgut
Source: BMC Genomics. 2015 Oct 28;16:871. doi: 10.1186/s12864-015-1981-7 (PMC4625525; doi:10.1186/s12864-015-1981-7)
Supplement: Additional file 11: — Transcriptome annotation pipeline. Data flow is shown by arrows and analytical steps by capital letters. (PDF 155 kb) [file 12864_2015_1981_MOESM11_ESM.pdf]

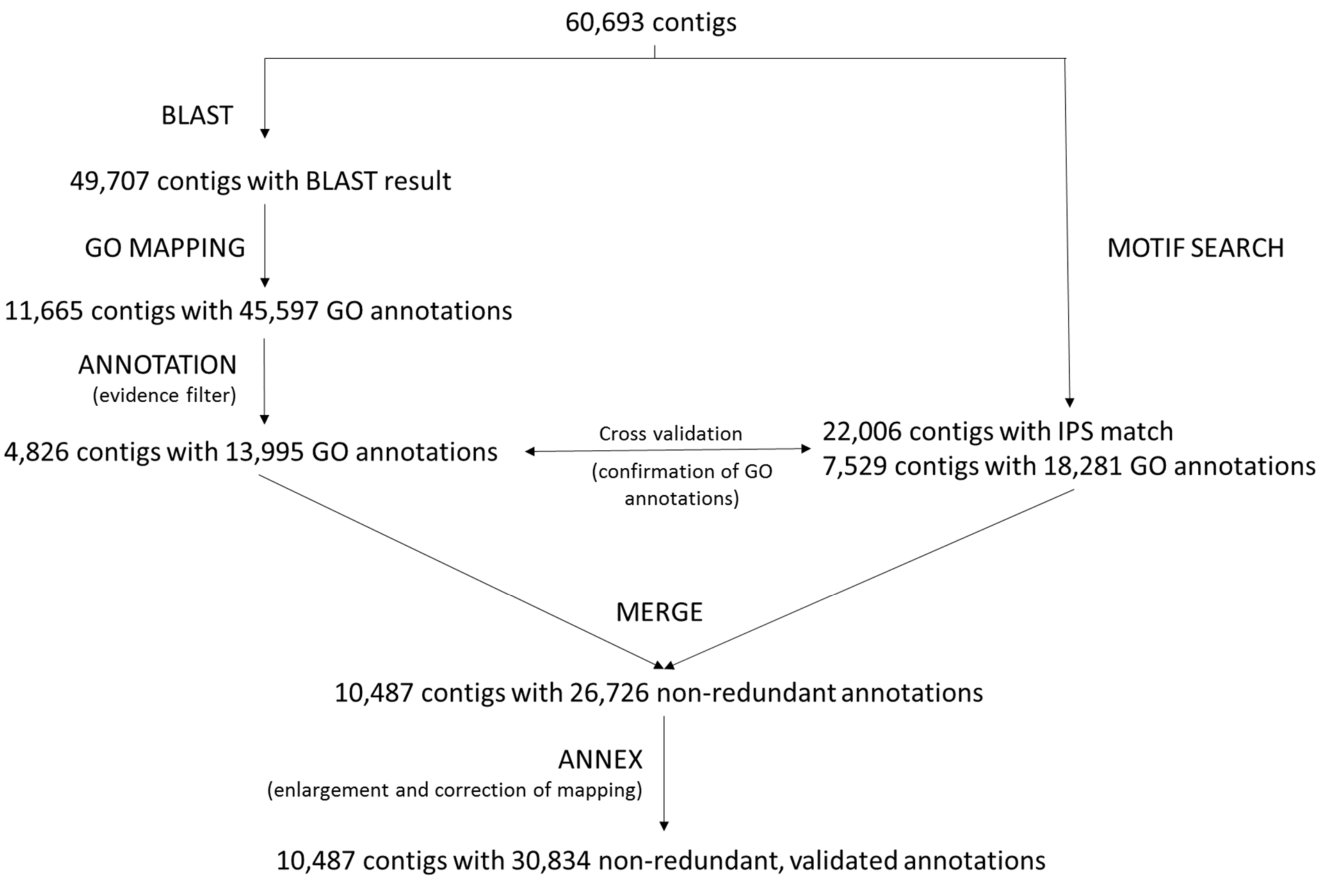

Additional file 10: Transcriptome annotation pipeline. Data flow is indicated by arrows and analysis steps by capital letters.
