# Supplementary material for: Integration of Ixodes ricinus genome sequencing with transcriptome and proteome annotation of the naïve midgut
Source: BMC Genomics. 2015 Oct 28;16:871. doi: 10.1186/s12864-015-1981-7 (PMC4625525; doi:10.1186/s12864-015-1981-7)
Supplement: Additional file 12: — Combined direct acyclic graphs for the different GO categories “molecular function” (A), “biological process” (B) and “cellular component” (C). (PDF 90 kb) [file 12864_2015_1981_MOESM12_ESM.pdf]

A

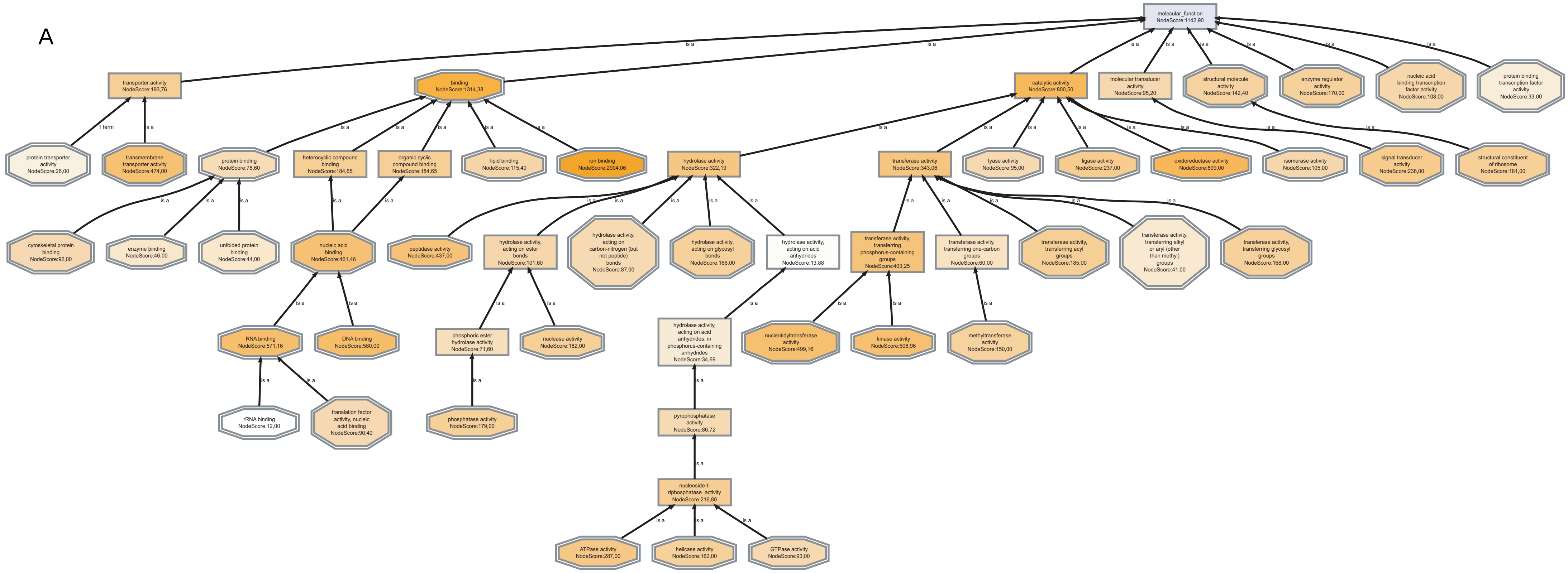

RNA-Seq all-1-1

B

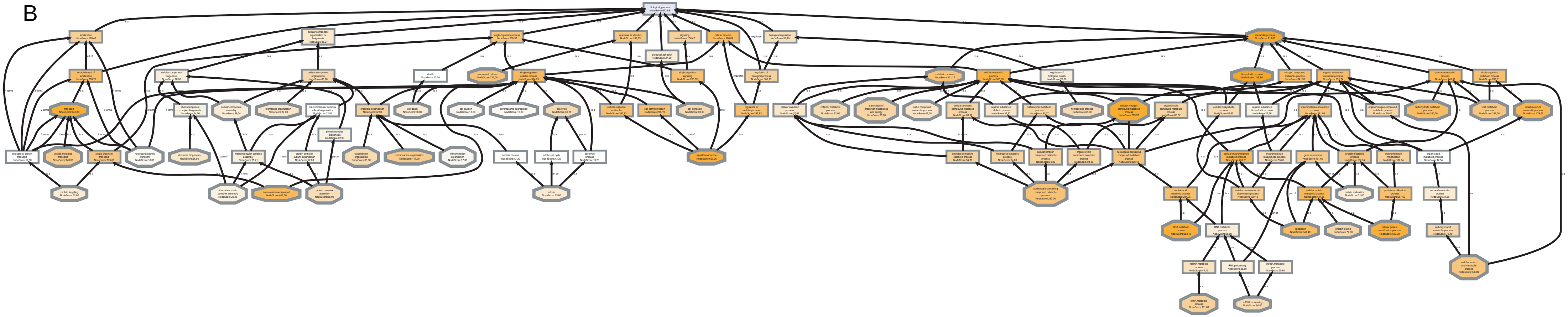

C

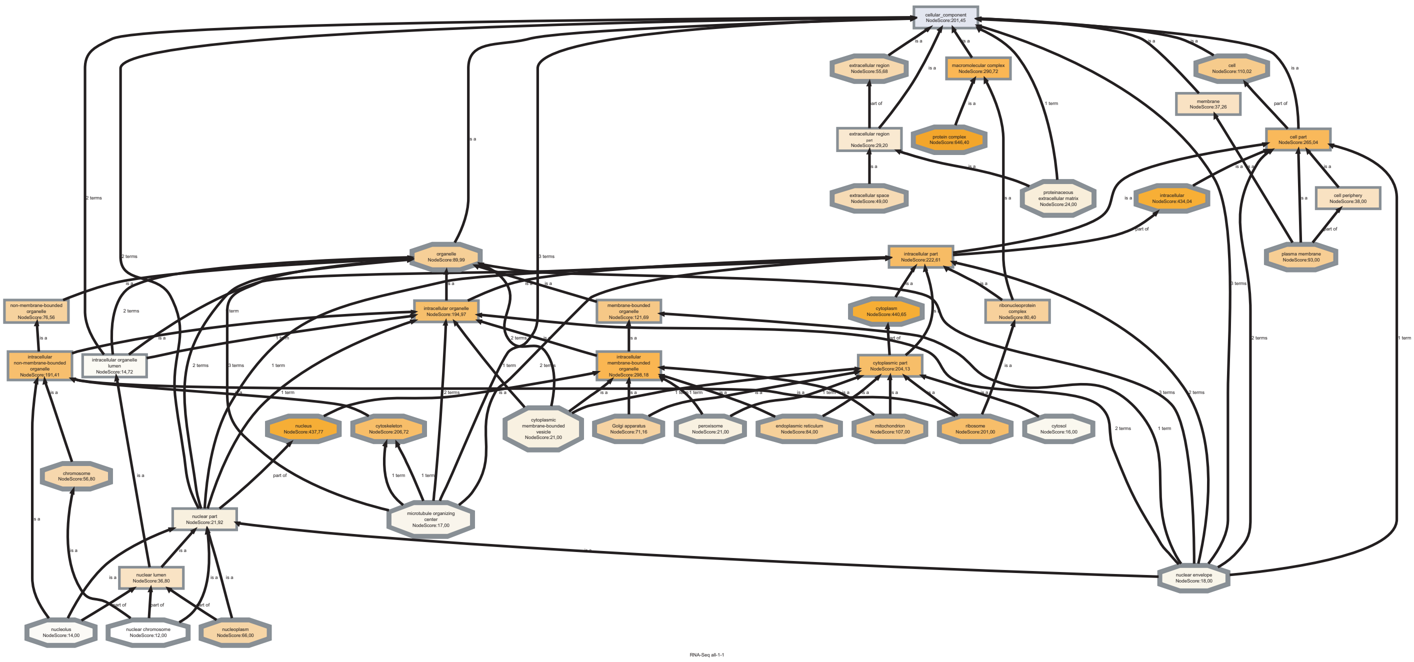

Additional file 11: Combined direct acyclic graphs for the GO categories "molecular function" (A), "biological process" (B) and "cellular component" (C)
